# Supplementary material for: Iron Deficiency and Reduced Muscle Strength in Patients with Acute and Chronic Ischemic Stroke
Source: J Clin Med. 2022 Jan 25;11(3):595. doi: 10.3390/jcm11030595 (PMC8836993; doi:10.3390/jcm11030595)
Supplement: Supplementary file 1 [file jcm-11-00595-s001.zip › jcm-1515622-supplementary.pdf]

Supplementary Table 1. Comparison in clinical characteristic of the entire cohort and follow-up (FU) cohort at baseline.

**Clinical Parameter**	**All patients N =140**	**FU cohort N=64**	**P**
Age, y, mean ± SD	69 ± 13	69 ± 11	n.s
Body mass index, kg/m², mean ± SD	27.7 ± 4.6	27.3 ± 3.9	n.s
Systolic blood pressure, mmHg, mean ± SD	136 ± 28	137 ± 30	n.s
Diastolic blood pressure, mmHg, mean ± SD	79 ± 14	81 ± 13	n.s
Mean blood pressure, mmHg, mean ± SD	68 ± 48	68± 33	n.s
Heart rate, beats/min, mean ± SD	69±13	71 ± 12	n.s
Female sex, N (%)	55 (39)	24 (38)	n.s
**Stroke etiology**			
Large-artery atherosclerosis, N (%)	49 (35)	25 (39)	n.s
Cardioembolic, N (%)	44 (31)	19 (30)	n.s
Small-vessel occlusion, N (%)	25 (18)	8 (12)	n.s
Stroke of undetermined etiology, N (%)	22 (16)	12 (19)	n.s
**Stroke severity**			
National Institute of Health Stroke Scale (NIHSS)			
Mean score ± SD	4.6 ± 3.5	4.2 ± 3.1	n.s.
0 - 4, N (%)	79 (59)	42 (66)	n.s
Modified Rankin Scale (mRS)			
Mean score ± SD	2.4 ± 1.5	2.0 ± 1.3	n.s.
0 - 1, N (%)	63 (47)	31 (48)	n.s

**Comorbidities**			
Diabetes mellitus, N (%)	40 (29)	18 (28)	n.s
Arterial hypertension, N (%)	96 (69)	48 (75)	n.s.
Dyslipidemia, N (%)	45 (32)	30 (47)	n.s.
Anemia, N (%)	25 (19)	11 (17)	n.s.
Cardiovascular disease, N (%)	58 (41)	27 (42)	n.s.
**Biochemistry**			
Hemoglobin, mg/dl, mean ± SD	14.0 ± 1.9	14.0 ± 2.0	n.s.
White blood cells count, mean ± SD	8.3 ± 2.5	9.2 ± 3.4	n.s.
Creatinine, mg/dl, mean ± SD	0.9 ± 0.4	1.0 ± 0.5	n.s.
Cholesterol, mg/dl, mean ± SD	187 ± 46	193 ± 45	n.s.
High density lipoprotein, mg/dl, mean ± SD	48.6 14.7	51.0 ± 15.3	n.s
Low density lipoprotein, mg/dl, mean ± SD	111.7 ± 40.0	113.4 ± 39.8	n.s.
Hemoglobin A1c, %, mean ± SD	6.1 ± 1.2	6.3 ± 1.4	n.s.
C-reactive protein, mg/l [IQR]	5.5 [1.7-11.8]	4.8 [1.6-10.1]	n.s.
Systemic inflammation, N (%)	63 (45)	24 (38)	n.s
**Medication**			
Antiplatelet drugs, N (%)	120 (86)	56 (88)	n.s
Anticoagulants, N (%)	29 (21)	12 (19)	n.s.
Proton pump inhibitors, N (%)	34 (24)	14 (22)	n.s.
β-blocker, N (%)	57 (41)	28 (44)	n.s.
ACE-inhibitors, N (%)	65 (46)	26 (41)	n.s
Ca-channel antagonists, N (%)	14 (10)	6 (9)	n.s.
Angiotensin receptor blockers, N (%)	4 (3)	3 (5)	n.s.

Diuretics, N (%)	29 (21)	13 (20)	n.s.
Statins, N (%)	100 (71)	48 (75)	n.s.

Supplementary Table 2. Baseline clinical characteristics of patients available to the clinical follow up examination one year after stroke according to presence of iron deficiency (ID) at follow up

Clinical Parameter	Normal Iron N =15	ID N =49	ID type I N =31	ID type II N =18	P value ID vs. normal iron	P value ID I + ID II vs. normal iron
Age, y, mean ± SD	65 ± 9	70 ± 12	69 ± 12	72 ± 11	n.s.	n.s.
Body mass index, kg/m², mean ± SD	27.0 ± 3.0	27.0 ± 3.0	27.4 ± 4.9	27.1 ± 2.4	n.s.	n.s.
Systolic blood pressure, mmHg, mean ± SD	137 ± 33	146 ± 18	136 ± 22	129 ± 19	n.s.	n.s.
Diastolic blood pressure, mmHg, mean ± SD	81 ± 13	84 ± 10	75 ± 16	74 ± 10	n.s.	n.s.
Heart rate, beats/min, mean ± SD	73 ± 12	77 ± 9	68 ± 9	79 ± 14	n.s.	n.s.
Female sex n, %	-	24 (49)	17 (55)	7 (39)	n.s.	n.s.
**Stroke severity**						
National Institute of Health Stroke Scale, mean ± SD	4.4 ± 3.7	4.4 ± 3.0	3.9 ± 2.8	5.2 ± 3.3	n.s	n.s
Modified Rankin Scale, mean ± SD	2.2 ± 1.3	1.9 ± 1.3	1.7 ± 1.1	2.3 ± 1.4	n.s.	n.s.
Self-reported appetite, mean ± SD	7.6 ± 1.6	6.4 ± 2.1*	6.7 ± 2.1	6.0 ± 2.2*	0.01	0.05
**Comorbidities**						
Diabetes mellitus, N (%)	4 (26)	16 (33)	9 (29)	5 (28)	n.s.	n.s.
Arterial hypertension, N (%)	11 (73)	37 (76)	24 (77)	13 (72)	n.s.	n.s.
Dyslipidemia, N (%)	8 (53)	23 (47)	15 (48)	7 (39)	n.s.	n.s.
Anemia, N (%)	1 (7)	16 (33)*	12 (39)	4 (22)	<0.5	n.s.
Cardiovascular disease, N (%)	8 (53)	19 (39)	12 (39)	7 (39)	n.s.	n.s.
**Medication**						

Antiplatelet drugs, N (%)	14 (93)	42 (86)	28 (90)	14 (78)	n.s.	n.s.
Anticoagulants, N (%)	2 (13)	10 (20)	5 (16)	5 (28)	n.s.	n.s.
Proton pump inhibitors	1 (7)	15 (31)	5 (16)	8 (44)	n.s.	n.s.
β-blocker, N (%)	6 (40)	22 (45)	15 (48)	7 (39)	n.s.	n.s.
ACE-inhibitors, N (%)	9 (60)	17 (35)	11 (35)	6 (33)	n.s.	n.s.
Ca-channel antagonists, N (%)	-	2 (11)	-	4 (29)	n.s.	n.s.
Angiotensin receptor blockers, N (%)	-	3 (6)	2 (6)	1 (6)	n.s.	n.s.
Diuretics, N (%)	2 (13)	11 (22)	6 (19)	5 (28)	n.s.	n.s.
Statins, N (%)	12 (80)	36 (74)	24 (77)	12 (66)	n.s.	n.s.
**Biochemistry**						
Hemoglobin, mg/dl, mean ± SD	14.8 ± 1.3	13.2 ± 1.9**	12.9 ± 1.7***	13.7 ± 2.0	<0.01	<0.01
White blood cells count, mean ± SD	7.0 ± 1.6	7.1 ± 2.2	7.1 ± 2.1	7.2 ± 2.4	n.s.	n.s.
Creatinine, mg/dl, mean ± SD	1.1 ± 0.3	1.0 ± 0.3	1.0 ± 0.3	0.9 ± 0.3	<0.05	n.s.
Cholesterol, mg/dl, mean ± SD	162 ± 26	174 ± 39	178 ± 40	166 ± 37	n.s.	n.s.
High density lipoprotein, mg/dl, mean ± SD	43 ± 9	57 ± 13***	57 ± 15***	56 ± 9**	<0.001	<0.01
Low density lipoprotein, mg/dl, mean ± SD	86 ± 25	93 ± 34	94 ± 35	90 ± 34	n.s.	n.s.
Hemoglobin A1c, %, mean ± SD	6.2 ± 0.6	6.4 ± 1.6	6.6 ± 1.5	6.0 ± 0.5	n.s.	n.s.
C-reactive protein, mg/l [IQR]	1.2 [1.0-3.4]	2.5 [1.0-6.2]	1.7 [1.0-5.9]	3.5 [1.0-8.7]*	n.s.	n.s.
Systemic inflammation, N (%)	1 (7)	12 (25)	7 (23)	5 (28)	n.s.	n.s.

***p<0.05 vs. Normal Iron; **<0.01 vs. Normal Iron, ***p<0.001 vs. Normal Iron**

Supplementary Table 3. Short physical performance battery (SPPB) at baseline in the study groups.

**SPPB Category**	**Normal iron status N =73**	**Iron deficiency N =65**	**P value**
SPPB attended, N (%)	36 (50)	34 (51)	n.s.
SPPB score, mean ± SD	9±3	8±3	n.s.
Balance test attended, N (%)	34 (47)	34 (52)	n.s.
Balance test score, mean ± SD	3±1	3±1	n.s.
Walk test attended, N (%)	32 (44)	27 (41)	n.s.
4-m gait speed, mean ± SD	1±0	1±0	n.s.
Chair stands attended, N (%)	32 (44)	29 (44)	n.s.
Chair stands score, mean ± SD	3±1	3±1	n.s.
